# Pridopidine Reverses Phencyclidine-Induced Memory Impairment

**DOI:** 10.3389/fphar.2018.00338

**Published:** 2018-04-10

**Authors:** Kristoffer Sahlholm, Marta Valle-León, Víctor Fernández-Dueñas, Francisco Ciruela

**Affiliations:** ^1^Unitat de Farmacologia, Departament Patologia i Terapèutica Experimental, Facultat de Medicina i Ciències de la Salut, IDIBELL-Universitat de Barcelona, L'Hospitalet de Llobregat, Barcelona, Spain; ^2^Department of Neuroscience, Karolinska Institutet, Stockholm, Sweden; ^3^Institut de Neurociències, Universitat de Barcelona, Barcelona, Spain

**Keywords:** dopamine stabilizers, cognition, mice, sigma-1 receptor, phencyclidine

## Abstract

Pridopidine is in clinical trials for Huntington's disease treatment. Originally developed as a dopamine D_2_ receptor (D_2_R) ligand, pridopidine displays about 100-fold higher affinity for the sigma-1 receptor (sigma-1R). Interestingly, pridopidine slows disease progression and improves motor function in Huntington's disease model mice and, in preliminarily reports, Huntington's disease patients. The present study examined the anti-amnesic potential of pridopidine. Thus, memory impairment was produced in mice by administration of phencyclidine (PCP, 10 mg/kg/day) for 10 days, followed by 14 days' treatment with pridopidine (6 mg/kg/day), or saline. Finally, novel object recognition performance was assessed in the animals. Mice receiving PCP and saline exhibited deficits in novel object recognition, as expected, while pridopidine treatment counteracted PCP-induced memory impairment. The effect of pridopidine was attenuated by co-administration of the sigma receptor antagonist, NE-100 (10 mg/kg). Our results suggest that pridopidine exerts anti-amnesic and potentially neuroprotective actions. These data provide new insights into the therapeutic potential of pridopidine as a pro-cognitive drug.

## Introduction

Pridopidine (formerly ACR16), was originally developed as a fast-dissociating, micromolar affinity dopamine D_2_ receptor (D_2_R) antagonist (Pettersson et al., 2010), showing antipsychotic-like activity in rodents with low liability to induce catalepsy (Nilsson et al., 2004; Natesan et al., 2006; Ponten et al., 2010) and pro-social effects in MK-801-treated rats (Rung et al., 2005). Later, pridopidine was found to possess nanomolar sigma-1 receptor (sigma-1R) affinity, displaying a corresponding preference for sigma-1R over D_2_R *in vivo* (Sahlholm et al., 2013, 2015). While showing promising effects on negative symptoms of schizophrenia in limited clinical studies (Carlsson and Carlsson, 2006), pridopidine subsequently underwent phase IIb and phase III trials in Huntington's disease patients, where improvements in motor function were observed (de Yebenes et al., 2011; Huntington Study Group HART Investigators, 2013). Additionally, preliminary results showed a slower decline in total functional capacity in Huntington's disease patients receiving pridopidine, suggesting a possible neuroprotective effect (Reilmann et al., 2017).

Pridopidine has also been found to increase brain-derived neurotrophic factor (BDNF) signaling, improve motor function and extend the lifespan of Huntington's disease model mice, and may exert Sigma-1R-dependent neuroprotective effects in neurons from such mice (Squitieri et al., 2015; Geva et al., 2016; Ryskamp et al., 2017). However, although neurodegenerative pathologies, including Huntington's disease, often feature cognitive deficits and memory impairment (Tyebji and Hannan, 2017), the putative effects of pridopidine on memory function have not yet been studied.

Sigma-1R agonists are known to exert anti-amnesic effects in several models of cognitive impairment (Maurice and Su, 2009). Novel object recognition has previously been used to demonstrate protective effects of atypical antipsychotics and sigma-1R agonists against cognitive deficits induced by subchronic phencyclidine (PCP) (Hashimoto et al., 2007). The subchronic PCP model is often used to model cognitive symptoms of schizophrenia (Rajagopal et al., 2014), but the neurotoxic effects of the treatment (Olney et al., 1989; Johnson et al., 1998) also makes this model relevant for studying neuroprotective effects. The present study aimed to investigate the ability of pridopidine to counteract novel object recognition deficits induced by subchronic PCP treatment in mice.

## Materials and methods

### Drugs

Pridopidine was custom-synthesized by Axon MedChem B.V. (Groningen, The Netherlands), while PCP and NE-100 were obtained from Tocris Bioscience (Bristol, UK).

### Animals

Wild type CD-1 mice (Charles River Laboratories) weighing 25–50 g were used at 2–3 months of age. The University of Barcelona Committee on Animal Use and Care approved the protocol. Animals were housed and tested in compliance with the guidelines described in the Guide for the Care and Use of Laboratory Animals (Clark et al., 1997) and following the European Union directives (2010/63/EU), FELASA, and ARRIVE guidelines. All efforts were made to minimize animal suffering and the number of animals used. Animals were housed in groups of five in standard cages with *ad-libitum* access to food and water and maintained under 12 h dark/light cycle (starting at 7:30 a.m.), 22°C temperature, and 66% humidity (standard conditions).

**Figure 1:**
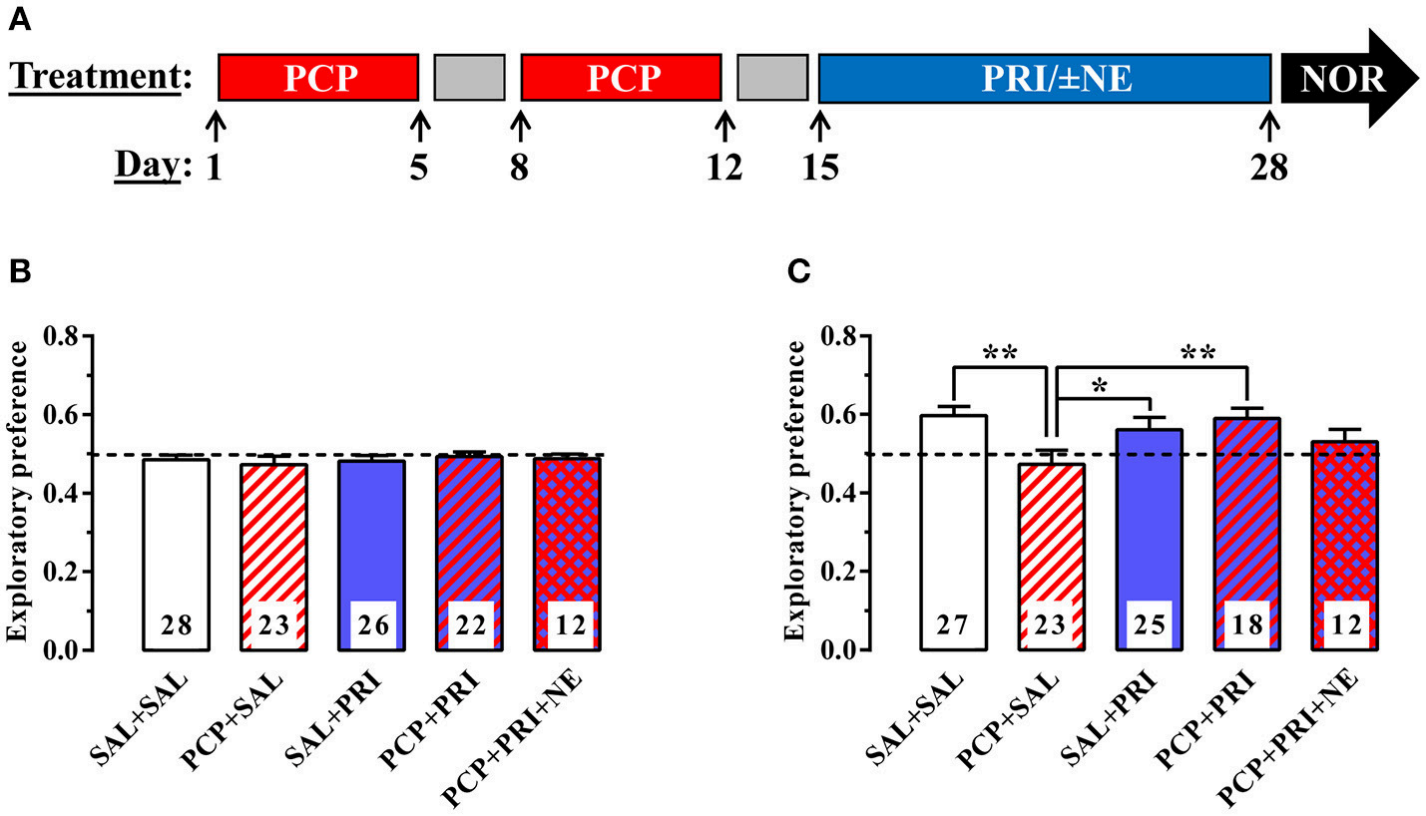
Effect of pridopidine on subchronic PCP-induced deficits in novel object recognition. Diagram of the drug treatment regime **(A)**. The exploratory preference for the rightmost of two identical objects was first assessed during the acquisition session **(B)**. The exploratory preference for the new object was evaluated during the retention trial **(C)**. The number of animals used per group is indicated on each column. **P < 0.01 and *P < 0.05 by one-way ANOVA with Bonferroni's multiple comparisons post-test. SAL, saline; PCP, phencyclidine; PRI, pridopidine; NE, NE-100; NOR, novel object recognition.

Animals received subcutaneous (s.c.) injections of PCP (10 mg/kg, 4 mg/ml), or saline (2.5 ml/kg), once daily for 10 days (on days 1–5 and 8–12), followed by intraperitoneal (i.p.) administration of either 6 mg/kg pridopidine (2 mg/ml), saline (3 ml/kg), or pridopidine + 10 mg/kg NE-100 (the injected solution contained both 2 mg/ml pridopidine and 3.33 mg/ml NE-100) once daily for another 2 consecutive weeks (days 15–28; Figure 1A).

### Novel object recognition

After completing the drug treatment regime, animals were habituated to the behavioral arena (a wooden box measuring 30 × 30 cm and painted non-reflective black, evenly illuminated at 110 lux) subsequently to be used for novel object recognition testing, by letting them explore the arena for 5 min on 3 consecutive days. Following the 3 days of habituation, two virtually identical objects were placed into the arena about 20 cm from each other, and the mice were allowed to explore the objects for 5 min, during which exploration of the objects was scored by the experimenter (acquisition session). Animals were considered to be exploring an object when sniffing or touching it with its forepaws while sniffing. Subsequently, 24 h after the acquisition session, animals were reintroduced to the arena, where one of the two objects from the acquisition session had been replaced with a new object, similar in size but with a distinct shape and color (retention session), and exploration was scored during 5 min as on the previous day. Two different types of objects were used in the study, and roughly half of the mice were trained on either type. Animals which failed to explore the objects for more than 6 s were excluded from the analysis. The ratio between time spent exploring the rightmost object (during the acquisition session), or the novel one (during the retention session), and total time spent exploring either object was calculated as a measure of cognitive performance.

### Statistical analysis

Data are represented as means ± S.E.M. Comparisons among experimental and control groups were performed by one-way analysis of variance (ANOVA), followed by Bonferroni-corrected post-tests. Statistical significance was accepted when *P* < 0.05.

## Results

We used the novel object recognition paradigm, considered to test the rodent equivalent of human declarative memory (Winters et al., 2010), to assess whether pridopidine can reverse subchronic PCP-induced deficits. During the acquisition session, all treatment groups showed undistinguishable preference for exploring the right or left object [*F*_(4, 106)_ = 0.2691, *P* = 0.8973] (Figure 1B). However, during the retention session, when one of the identical objects from the acquisition session was replaced by a novel object, the different treatment groups showed significant differences [*F*_(4, 100)_ = 2.932, *P* = 0.0244]. Interestingly, mice treated with PCP + saline showed significantly reduced preference (*P* < 0.01) for the novel object compared to control animals treated with saline + saline (Figure 1C), as previously described (Hashimoto et al., 2007). Importantly, PCP-treated animals that received 6 mg/kg pridopidine daily during the last 14 days showed a significantly greater preference (*P* < 0.05) for the novel object compared to the PCP + saline group. Mice receiving saline + pridopidine treatment did not differ significantly from the saline + saline group (Figure 1C).

Finally, we aimed to assess the putative sigma receptor involvement in the pridopidine-mediated reversion of PCP-induced deficits in novel object recognition. The sigma-1R-preferring antagonist, NE-100 (10 mg/kg), attenuated the effect of pridopidine, such that the exploratory preference of NE-100-treated animals was not significantly different (*P* = 0.255) from that of animals receiving PCP + saline (Figure 1C).

## Discussion

The present investigation provided evidence that pridopidine mediates pro-cognitive effects, counteracting subchronic PCP-induced deficits in novel object recognition in mice. We used 6 mg/kg pridopidine as this dose was found to confer neuroprotection in a Huntington's disease mouse model (Squitieri et al., 2015). PCP is known to induce neurotoxic histopathological changes when given acutely (Olney et al., 1989) and to cause neuronal apoptosis when administered subchronically to rodents (Johnson et al., 1998). Thus, the ability of pridopidine to reverse subchronic PCP-induced novel object recognition deficits might reflect the neuroprotective effects of this ligand described by recent studies (Squitieri et al., 2015; Geva et al., 2016; Garcia-Miralles et al., 2017; Ryskamp et al., 2017). Protection from PCP-induced deficits in novel object recognition by sigma-1R agonist treatment has been suggested to be mediated via sigma-1R-induced increased BDNF secretion, and was abolished by simultaneous treatment with the sigma-1R-preferring antagonist, NE-100 (Hashimoto et al., 2007). Interestingly, pridopidine has been shown to increase BDNF expression *in vivo* (Squitieri et al., 2015) and in a neuroblastoma cell line (Geva et al., 2016); the latter effect was also blocked by NE-100. Similarly, in the present study, NE-100 counteracted the effect of pridopidine to reverse PCP-induced deficits in novel object recognition.

It should be mentioned that although NE-100 is 200-fold selective for sigma-1R over sigma-2 biding sites (Berardi et al., 2001), engagement of sigma-2 receptors, or other “secondary” targets of NE-100, cannot be ruled in the present context, given the high dose of NE-100 employed. Thus, while it is tempting to speculate that sigma-1R engagement may be responsible for the anti-amnesic effects of pridopidine observed here, further studies will be necessary to substantiate this notion.

Overall, the present results suggest that pridopidine exerts anti-amnesic effects in subchronically PCP-treated mice, which may be relevant to the therapeutic potential of this compound not only in Huntington's disease (e.g., slowing functional decline) but also in other pathological conditions characterized by cognitive impairment.

## Author contributions

KS: designed and performed *in vivo* experiments, analyzed data, and wrote the paper; MV-L: performed *in vivo* experiments; VF-D: designed experiments and wrote the paper; FC: supervised the project, designed experiments, and wrote the paper.

### Conflict of interest statement

The authors declare that the research was conducted in the absence of any commercial or financial relationships that could be construed as a potential conflict of interest. The reviewer DB declared a shared affiliation, though no other collaboration, with one of the authors KS to the handling Editor.
